# Retrieval of B1 phase from high-pressure B2 phase for CdO nanoparticles by electronic excitations in Cd*_x_*Zn_1−_*_x_*O composite thin films

**DOI:** 10.3762/bjnano.16.43

**Published:** 2025-04-17

**Authors:** Arkaprava Das, Marcin Zając, Carla Bittencourt

**Affiliations:** 1 Chimie des Interaction Plasma surface, Research Institute for Materials Science and Engineering, Université of Mons, 7000 Mons, Belgium; 2 SOLARIS National Synchrotron Radiation Centre, Jagiellonian University, 30-392 Krakow, Poland

**Keywords:** irradiation, phase transformation, thermal spike, track diameter, X-ray absorption near edge spectroscopy, X-ray photoelectron spectroscopy

## Abstract

This study investigates the recovery of the B1 phase from the high-pressure B2 phase, at atmospheric pressure, in cadmium oxide (CdO) nanoparticles incorporated within sol–gel synthesized Cd*_x_*Zn_1−_*_x_*O (*x* = 0.40) composite thin films. The recovery process is investigated using electronic excitations as an effective tool. Exposure to 120 MeV silver ion irradiation results in the complete amorphization of the B2 phase in CdO nanoparticles, while the crystalline hexagonal wurtzite phase of zinc oxide (ZnO) remains intact. In contrast, 80 MeV oxygen ion irradiation preserves the B2 phase and facilitates the reemergence of the B1 phase. The partial damage caused by electronic energy loss during oxygen ion irradiation in the willemite Zn_2_SiO_4_ phase is identified as a trigger for the B1 to B2 phase transformation in CdO nanoparticles, enabling the recovery of the B1 phase. The diminishing local pressure exerted by the Zn_2_SiO_4_ phase on CdO nanoparticles during oxygen ion irradiation leads to the coexistence of both B1 and B2 phases. X-ray absorption near-edge spectra (XANES) reveal minimal changes in the intensity of the spike-like Zn *L*_3,2_ pre-edge feature associated with the Zn_2_SiO_4_ phase under oxygen ion irradiation, while it entirely disappears with silver ion irradiation, confirming the amorphization of the Zn_2_SiO_4_ phase. Complementary observations from X-ray photoelectron spectroscopy (XPS), specifically O 1s and Si 2p peaks in XPS spectra, support these findings. Additionally, the track diameter in CdO subjected to 120 MeV silver ion irradiation is calculated to be approximately 8 nm using an inelastic thermal spike simulation code. This study elucidates the intriguing reappearance of the B1 phase under oxygen ion irradiation and highlights the radiation stability of the B2 phase through diverse characterization techniques, demonstrating the potential reversibility of the B1 to B2 phase transformation induced by ion irradiation.

## Introduction

Zinc oxide (ZnO)-based thin films are of significant interest due to their wide bandgap value (3.37 eV at room temperature), transparent electrical conduction, and large excitonic binding energy (60 meV) [1]. In contrast, cadmium oxide (CdO) exhibits a lower bandgap of 2.2 eV, along with high electron mobility (>100 cm^2^/V/s) and high electrical conductivity (>10^14^ S/cm), demonstrating its potential for optoelectronic applications [2–4]. The incorporation of cadmium into ZnO effectively reduces the bandgap, rendering the thin films suitable for applications in the visible region of the electromagnetic spectrum [5]. Composite semiconducting thin films have garnered significant attention as their bandgap can be lowered without compromising mobility and conductivity. Beyond optoelectronic applications, CdO–ZnO-based alloys are also employed in gas-sensing technologies [6]. In prior investigations, we reported a local pressure-driven structural phase transformation (PT) from B1 (NaCl) to B2 (CsCl) in Cd*_x_*Zn_1−_*_x_*O (*x* = 0.4) composite binary oxide thin films [1]. The radiation stability of these phases is crucial for optoelectronic applications in space, where the exposure to high-energy particles and gamma radiation can induce lattice defects and lower the device efficiency [7]. Swift heavy ion (SHI) irradiation experiments provide valuable insights into the radiation stability of the transformed B2 phase, which is essential for the future utilization of these composite materials in space-based optoelectronic applications [7]. High-energy ion irradiation can lead to latent track formation or phase transitions, either from crystalline to crystalline or crystalline to amorphous, depending on the threshold electronic energy loss (*S*_eth_) [8–9]. The formation of latent tracks has been described through two primary models: the Coulomb explosion model, which relies on electrostatic repulsive forces [10–11], and the thermal spike model, where energy is transferred to lattice atoms, resulting in melting and subsequent quenching to form tracks [12–13]. The latter model has been more widely supported in the literature. Upon penetration in a solid, energetic ions lose energy through two mechanisms: direct energy transfer to target nuclei via elastic collisions (nuclear energy loss (*S*_n_)) and ionization of the target atoms through inelastic collisions (electronic energy loss (*S*_e_)). In the present investigation, we primarily focus on *S*_e_, given the high energy of the irradiation ions (several MeV), while *S*_n_ becomes more significant in the keV range [7].

Here, we report on the radiation stability of the transformed B2 phase under 120 MeV Ag and 80 MeV O ion irradiation. Notably, such an investigation has not been previously conducted, as the B2 phase has only been reported through hydrostatic pressure techniques using the in situ diamond anvil cell (DAC) technique [14]. Our study is facilitated by the successful achievement of the B2 phase at atmospheric pressure for Cd*_x_*Zn_1−_*_x_*O (*x* = 0.40) composite thin films [1]. Das et al. reported that *S*_eth_ for the formation of an amorphous latent track in rock salt CdO is 14.56 keV/nm [7]. Thus, we selected 120 MeV Ag ions, where electronic energy loss (*S*_e_) in CdO is 25.10 keV/nm, and 80 MeV O ions, with *S*_e_ at 1.9 keV/nm, to examine the effects on both sides of *S*_eth_ (i.e., higher and lower sides). Notably, the reappearance of the B1 phase was observed with O ion irradiation, while no such retrieval occurred with Ag ion irradiation. This intriguing finding is explored in detail in this investigation. Crystallographic characterization of both pristine and irradiated thin films was performed with X-ray diffraction and Raman spectroscopy. Additionally, X-ray absorption near-edge structure (XANES) spectroscopy was conducted at the Zn *L*_3,2_ and O *K* edges for all the thin films. X-ray photoelectron spectroscopy (XPS) on Si 2p and O 1s core levels provided direct evidence of changes in surface chemical states due to irradiation. In summary, we elucidate the underlying mechanism responsible for the retrieval of the B1 phase from the transformed B2 phase following O ion irradiation using core-level spectroscopy. While the B1 to B2 phase transformation is reported to be irreversible, our findings suggest that with the appropriate choice of irradiating ions and energies, the recovery of the B1 phase is possible. Complementary to this experimental investigation, we conducted thermal spike calculations with 120 MeV Ag ions in rock salt CdO.

## Experimental

Thin films with 40% cadmium concentration in zinc oxide (ZnO) were synthesized using the sol–gel chemical route method, as detailed in our previous publication [1]. The irradiation experiments were performed using the 15 UD tandem Pelletron accelerators at the Inter-University Accelerator Centre (IUAC), New Delhi. The irradiation was performed with 120 MeV Ag and 80 MeV O ions. Two different fluences were used for each ion type (i.e., 1 × 10^13^ ions/cm^2^ and 3 × 10^13^ ions/cm^2^). The focus of this study is on a film that underwent a complete transformation to the B2 phase, which is designated as a pristine thin film prior to further irradiation.

The characterization of the thin films was performed using X-ray diffraction (XRD) on a Bruker high-resolution X-ray diffractometer, employing a Cu Kα beam over a 2θ range of 30–50°. Raman spectroscopic measurements were conducted at room temperature with a SENTERRA spectrometer (Bruker), equipped with an Ar ion laser (532 nm) with 0.2 mW laser operating power. Scanning electron microscopy (SEM) analysis was carried out with a HITACHI SU8020 model, using an electron beam energy of 3.0 keV. X-ray photoelectron spectroscopy (XPS) was performed using an ESCA-5000 Versa Probe system (Physical Electronics) with an Al Kα (1486.7 eV) beam and a 124 mm hemispherical electron analyzer. X-ray absorption near-edge structure experiments were performed at PIRX beamline [15] in the SOLARIS synchrotron facility in Poland [16], focusing on Zn *L*_2,3_ and O *K* edges in total electron yield mode. Table 1 summarizes the irradiation ion energies and fluences for each thin film, along with their corresponding labels.

**Table 1:** Sample name with irradiating ion and fluence.

Sample acronym^a^	Post-synthesis annealing temperature (°C)	Irradiating ion and energy (MeV)	Irradiation fluence (ions/cm^2^)

Z700	700	no irradiation	0
Z900	900	no irradiation	0
CZ900_Pris	900	no irradiation	0
CZ900_113O	900	80 MeV O ion	1 × 10^13^
CZ900_313O	900	80 MeV O ion	3 × 10^13^
CZ900_113Ag	900	120 MeV Ag ion	1 × 10^13^
CZ900_313Ag	700	120 MeV Ag ion	3 × 10^13^

^a^Cd*_x_*Zn_1−_*_x_*O (*x* = 0.40) labelled CZ and ZnO labelled Z.

## Results and Discussion

### Influence of Ag and O ion irradiation on crystallographic phase by X-ray diffraction and Raman spectroscopy

**Figure 1:**
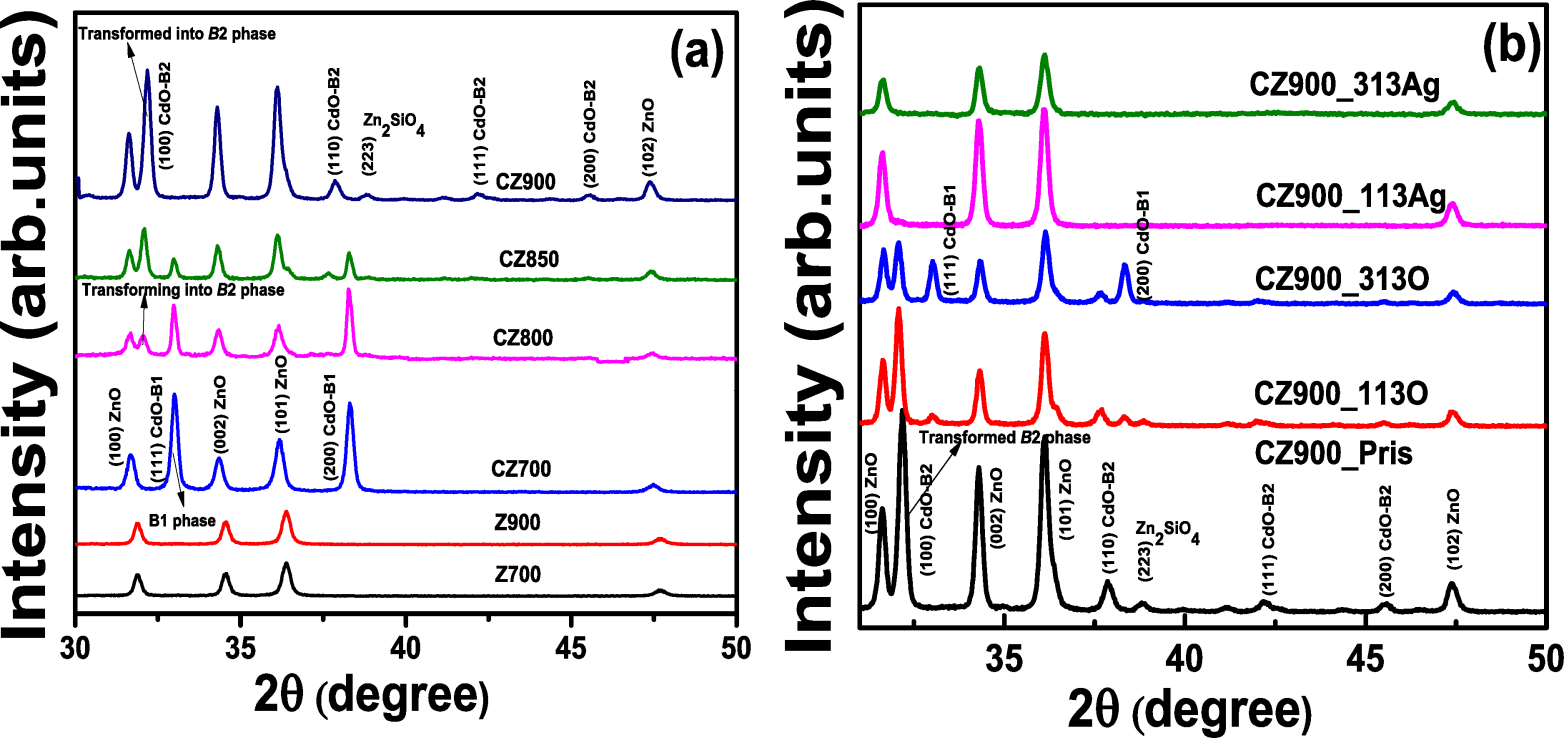
(a) Figure 1a was reprinted from [1], Acta Materialia, vol. 267, by A. Das; C. Latouche; S. Jobic; E. Gautron; A. Merabet; M. Zajac; A. Shibui; P. Krüger; W.-H. Huang; C.-L. Chen; A. Kandasami and C. Bittencourt, "Stabilization of the high-pressure phase of CdO by nanoparticle formation in Cd*_x_*Zn_1−_*_x_*O thin films", article no. 119744, Copyright (2024), with permission from Elsevier. This content is not subject to CC BY 4.0 (b) XRD pattern for CZ900_Pris, CZ900_113O, CZ900_313O, CZ900_113Ag, and CZ900_313Ag thin films.

Figure 1a shows the XRD patterns illustrating the B1 to B2 PT in Cd*_x_*Zn_1−_*_x_*O (*x* = 0.4) binary oxide thin films subjected to various annealing temperatures of 700, 800, 850, and 900 °C [1]. The data reveals the emergence of the B2 phase characterized by the (100) Bragg reflection in CdO nanoparticles as the annealing temperature increases. In contrast, the ZnO nanoparticles consistently exhibit the hexagonal wurtzite phase. Figure 1b portrays the effect of 80 MeV O and 120 MeV Ag ion irradiation on the CZ900_Pris sample, wherein the complete transition from B1 to B2 PT has already occurred. The reflections corresponding to the hexagonal wurtzite phase (space group *P*6_3_*mc*) are situated at 31.6° (100), 34.3° (002), 36.1° (101), and 47.4° (102) for the CZ900_Pris sample. The (100) and (110) Bragg reflections associated with the transformed B2 phase (space group *Pm*_−3_*m*) are located at 32.2° and 37.8°, respectively. Additionally, a low-intensity peak corresponding to willemite Zn_2_SiO_4_ (space group *R*_−3_) is observed at 38.8°, previously identified as a contributing factor to the B2 phase transition [1]. In the case of the CZ900_113O sample, a reduction in the intensity of the B2 phase reflections is noted, accompanied by a subtle emergence of the B1 phase (space group *Fm*_−3_*m*) at 32.9° and 38.3°, corresponding to the (111) and (200) reflections, respectively. The presence of the willemite Zn_2_SiO_4_ phase persists in the CZ900_113O sample. Upon applying an O ion irradiation fluence of 3 × 10^13^ ions/cm^2^ in the CZ900_313O sample, a further decrease in the intensity of the B2 phase is observed, which is attributed to enhanced electronic energy loss and the phenomenon of multiple ion impacts. The B1 phase is fully reestablished in this thin film, while the signature of the Zn_2_SiO_4_ phase is no longer detectable. This amorphization of the Zn_2_SiO_4_ phase facilitates the re-emergence of the B1 phase under higher fluence. For the CZ900_113Ag sample subjected to Ag irradiation, both the B2 phase and the Zn_2_SiO_4_ phase exhibit complete amorphization due to irradiation-induced damage, precluding any observable evolution of the B1 phase. Consequently, the wurtzite ZnO phase remains intact in both the CZ900_113Ag and CZ900_313Ag thin films. Notably, the absence of a distinct phase for CdO nanoparticles indicates that the radiation stability of the wurtzite ZnO structure significantly surpasses that of the B1/B2 CdO phase.

**Figure 2:**
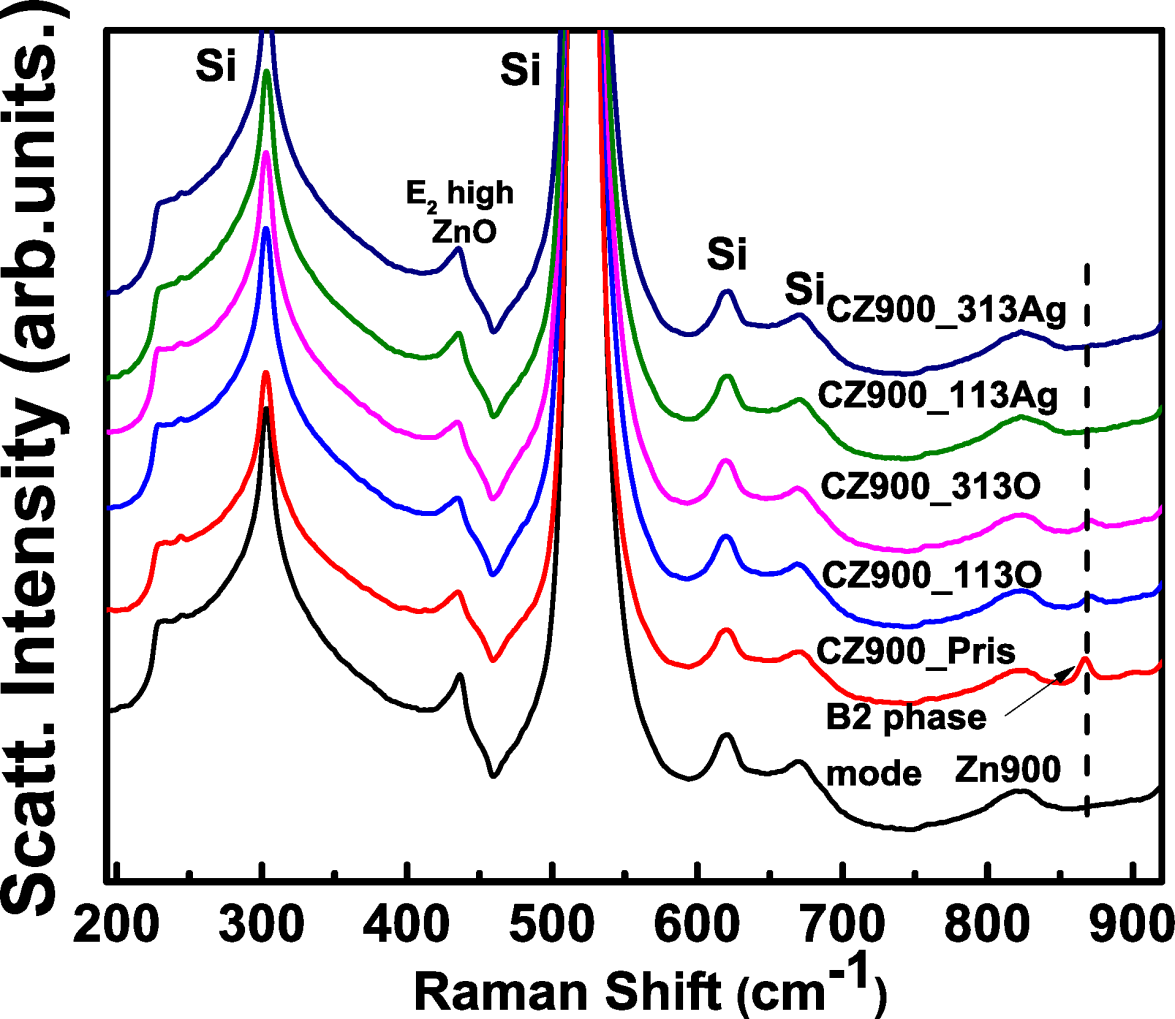
Raman spectra for undoped ZnO (Zn900), CZ900_Pris, CZ900_113O, CZ900_313O, CZ900_113Ag, and CZ900_313Ag thin films.

In Figure 2, the Raman spectra for both pristine and irradiated thin films are shown. The spectra for the undoped ZnO thin film annealed at 900 °C (Z900) are included to facilitate a comparative analysis concerning the presence of the B2 phase. These measurements were conducted in backscattering geometry without accounting for any polarization effects of the incident laser light. The optically active phonon modes at the center of the Brillouin zone have the following point symmetries:


[1] $$\Gamma_{\text{opt}}=1{\text{A}}_{1}+2{\text{B}}_{1}+1{\text{E}}_{1}+2{\text{E}}_{2}.$$

In this equation, the A_1_ and E_1_ modes correspond to Raman and infrared (IR) active branches, characterized by polar symmetries that further degenerate doubly longitudinal optical (LO) and transverse optical (TO) components with different frequencies. The E_2_ mode represents the only Raman-active nonpolar branch, which comprises two sub-branches, E_2_(H) and E_2_(L). This branch is both Raman and IR active [17]. The peaks observed in the Raman spectra are predominantly attributed to the silicon substrate, with notable peaks at 303, 520, 620, and 671 cm^−1^. The peak at 435.9 cm^−1^ corresponds to the E_2_(H) mode characteristic of the wurtzite ZnO phase [17]. The persistence of the E_2_(high) mode across all O and Ag ion irradiated thin films, indicates the stability of the wurtzite phase under the used irradiation conditions.

In Figure 2, the Raman spectra for CZ900_Pris and Z900 thin film are reproduced from our previous publication [1]. The presence of the B2 phase at 868.5 cm^−1^ is indicated by an arrow for the CZ900_Pris sample, which is absent in the Z900 sample. The intensity of this peak diminishes in the CZ900_113O and CZ900_313O samples, while it completely vanishes in the CZ900_113Ag and CZ900_313Ag samples. Thus, the Raman spectroscopic results, in conjunction with the XRD patterns, corroborate the observations regarding the presence of the B2 phase of CdO nanoparticles.

### Microscopic modifications observed from SEM micrographs

**Figure 3:**
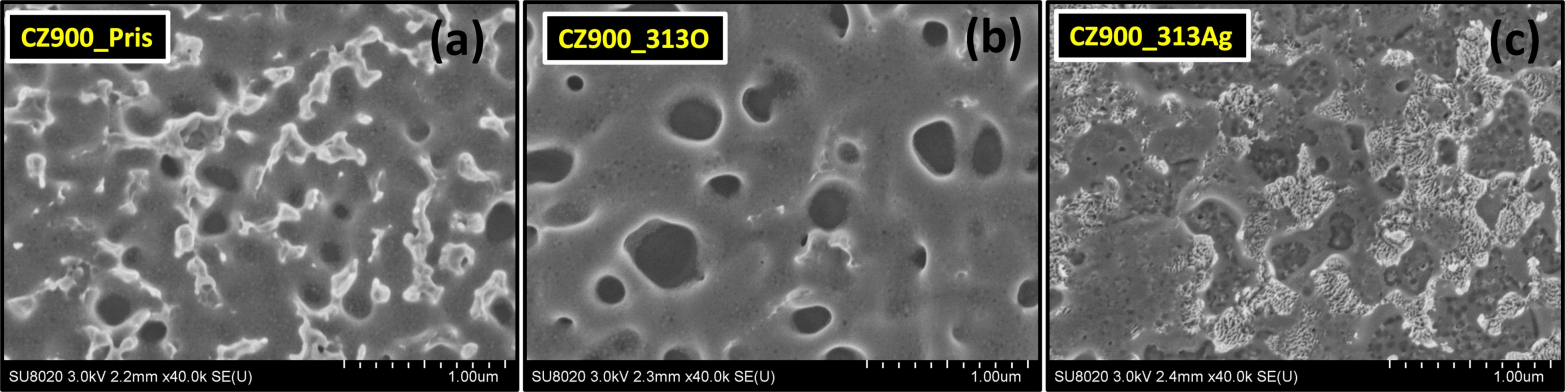
Plane view scanning electron microscopic images for CZ900_Pris (a), CZ900_313O (b), and CZ900_313Ag (c) thin films.

Figure 3a–c illustrates the SEM micrographs for the CZ900_Pris, CZ900_313O, and CZ900_313Ag thin films, respectively. In the CZ900_Pris sample, an interconnected homogenous distribution of grains is not evident; rather, distinct void regions are observed. Given that CdO has a melting temperature of ≈1000 °C, a portion of CdO may have melted during the annealing process at 900 °C, subsequently condensing in energetically favorable sites at the film surface. The whitish regions in the CZ900_Pris thin film may indicate areas enriched in cadmium. Our earlier investigation into CdO thin film noted similar Cd-rich whitish regions and the formation of nanosheets [18]. In the CZ900_313O thin film, no significant surface alterations were detected. However, the CZ900_313Ag thin film exhibits visible damage on the surface resulting from Ag ion irradiation. The previously distinct void regions are no longer present, suggesting that material may have been sputtered from the film surface due to Ag ion irradiation, potentially leading to the amorphization of the crystallographic B2 phase associated with CdO.

### Influence of ion irradiation on electronic structure from XANES spectra

**Figure 4:**
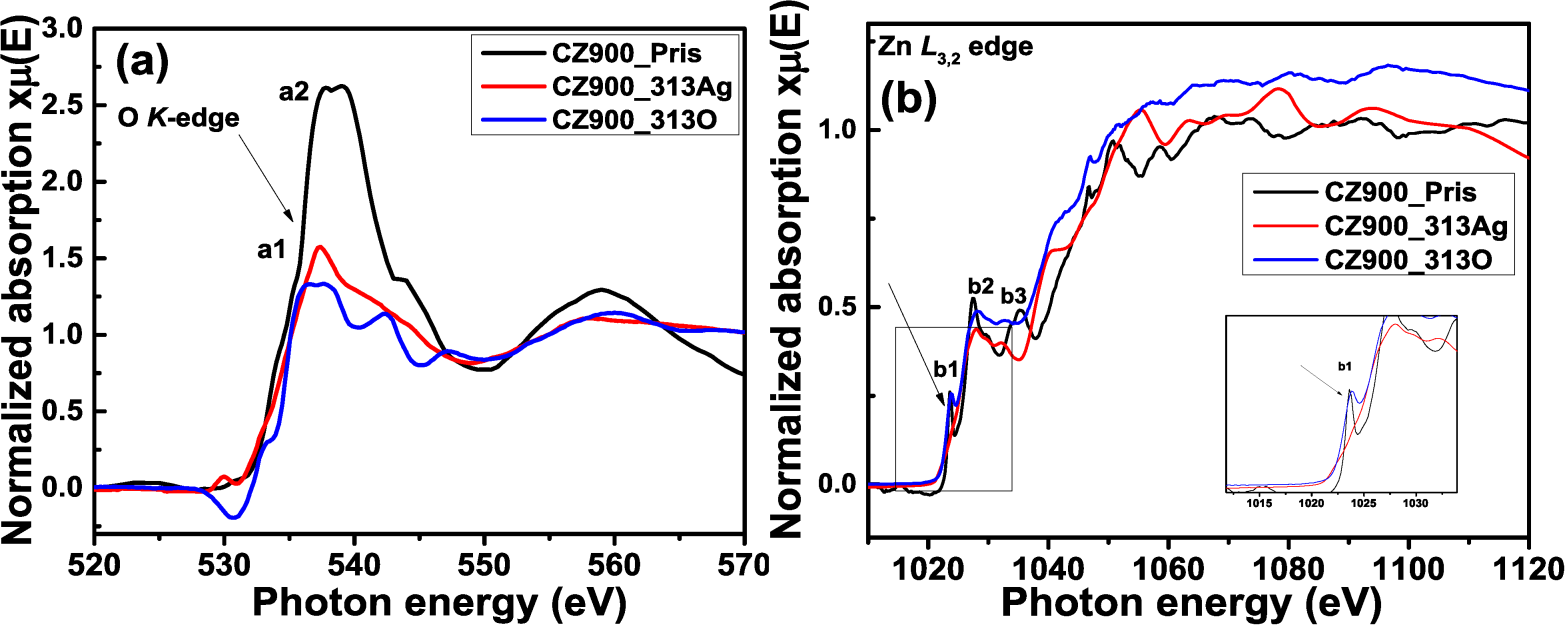
O *K* (a) and Zn *L*_3,2_ (b) XANES spectra for CZ900_Pris, CZ900_313O, and Z900_313Ag thin films.

Figure 4a,b presents the XANES spectra at the O *K* and Zn *L*_3,2_ edges for the CZ900_Pris, CZ900_313O, and CZ900_313Ag thin films. The spectra for the O *K* and Zn *L*_3,2_ edge for the CZ900_Pris sample are reproduced from our previous publication [1]. The prominent features a1 and a2 observed at 535 eV and 537 eV, respectively, in the O *K* edge spectrum of the CZ900_Pris sample correspond to electronic transitions from O 1s to hybridized Zn 4s and O 2p orbitals. In the CZ900_313O thin film, there is a significant reduction in the intensity of the O *K* edge spectrum. This attenuation suggests that O ion irradiation diminishes the likelihood of core-level electronic transitions from O 1s to the hybridized Zn 4s–O 2p orbitals. It is plausible that O ion irradiation generates oxygen vacancies (V_O_) at lattice sites and introduces defects such as interstitial oxygen (O_i_). The formation of V_O_ may hinder the hybridization between Zn 4s–O 2p orbitals, further decreasing the probability of electronic transitions, which is reflected in the diminished a1 and a2 features. In contrast, the spectral characteristics of the CZ900_313Ag sample exhibit a complete transformation compared to those of the CZ900_Pris sample. The introduction of a substantial number of defects may have relaxed the dipole selection rule, resulting in a destructive interference pattern within the multiple scattering signal. Such out-of-phase oscillations can lead to flat pre- and post-absorption edge features, lacking the typical wavy nature. However, direct evidence for the degradation of the Zn_2_SiO_4_ phase cannot be conclusively determined from the O *K* edge spectrum; this information is more clearly discernible from the Zn *L*_3,2_ edge, as illustrated in Figure 4b.

The main absorption edge in the Zn *L*_3,2_ edge begins at 1023 eV, and is associated with electronic transitions from the Zn 2p level to unoccupied Zn 4s states. Additionally, transitions from the Zn 2p_1/2_ → 3d antibonding orbitals significantly contribute to the formation of this main absorption edge. The spike-like feature designated as B1 arises from the formation of the willemite Zn_2_SiO_4_ phase [1]. Notable differences are observed in the b2 and b3 features of the CZ900_313Ag sample compared to the CZ900_Pris sample. Specifically, the B1 feature is absent in the CZ900_313Ag sample, providing clear evidence of the amorphization of the Zn_2_SiO_4_ phase due to Ag ion irradiation. In contrast, in CZ900_313O, the thin film exhibits a broader B1 feature with a larger full width at half maximum (FWHM) and reduced sharpness, although it does not completely disappear as seen in the CZ900_313Ag thin film. This observation further supports the presence of the Zn_2_SiO_4_ phase in the CZ900_313O thin film, which has not been detected in the XRD pattern due to limitations in detection sensitivity. Thus, X-ray absorption spectroscopy (XAS) has effectively addressed this limitation, providing evidence for the existence of the Zn_2_SiO_4_ phase. Furthermore, the presence of the Zn_2_SiO_4_ phase in the CZ900_313O thin film suggests local pressure conditions that may contribute to the formation of the B2 phase in CdO nanoparticles. Consequently, both XRD and XANES analyses yield complementary evidence regarding the presence of the B2 phase. In the Zn *L*_3,2_ edge (inset of Figure 4b), the B1 feature is magnified, clearly illustrating the changes in the B1 feature corresponding to the willemite Zn_2_SiO_4_ phase across all three thin films.

### Influence of irradiation on Zn_2_SiO_4_ phase from Si 2p XPS spectra

**Figure 5:**
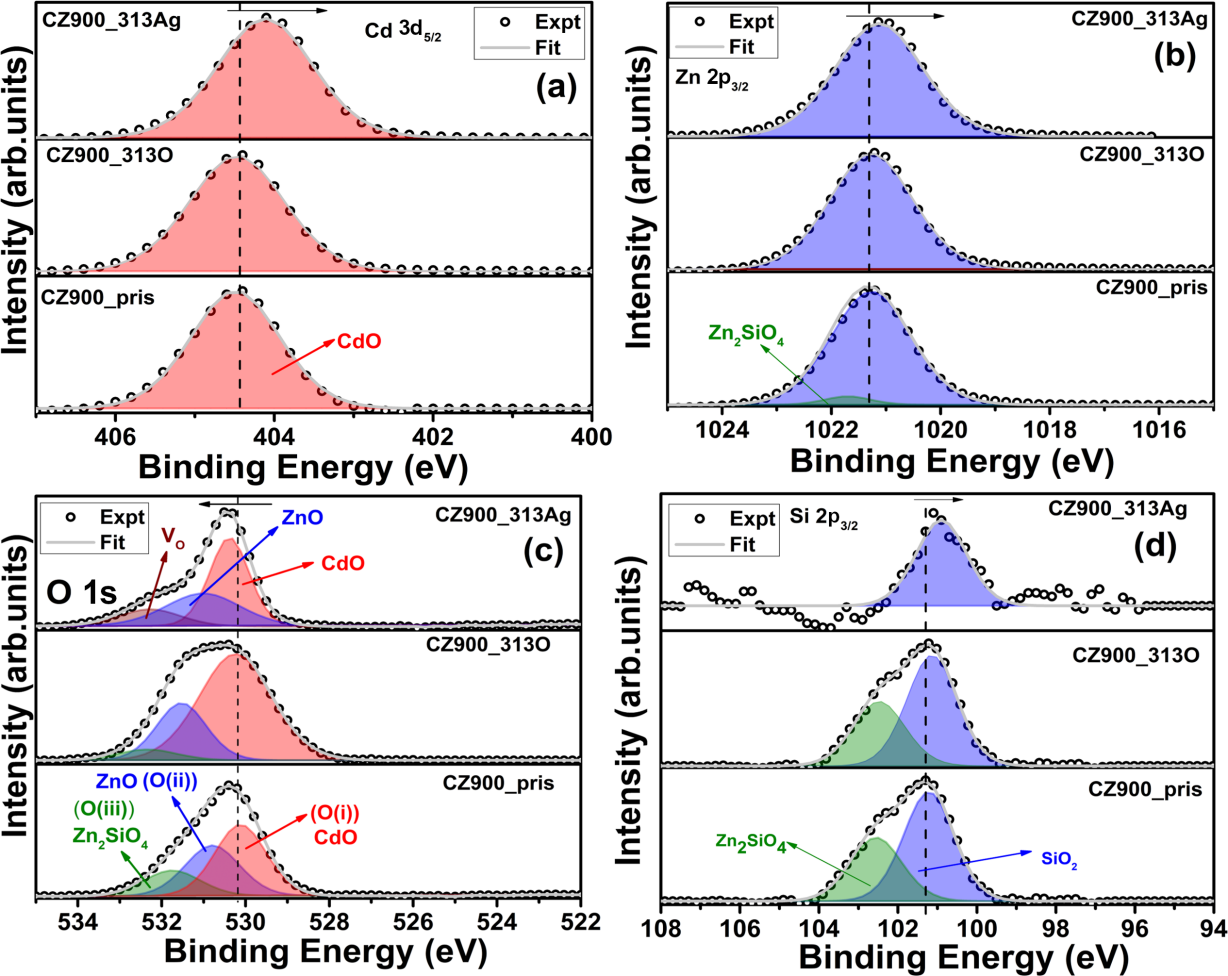
X-ray photoelectron spectra for Cd 3d_5/2_ (a), Zn 2p_3/2_ (b), O 1s (c), and Si 2p_3/2_ (d) edges for CZ900_Pris, CZ900_313O, and CZ900_313Ag thin films.

Figure 5a–d illustrates the Cd 3d_5/2_, Zn 2p_3/2_, O 1s, and Si 2p_3/2_ peaks in XPS spectra for both pristine and irradiated thin films. The calibration of all peaks was conducted using the C 1s peak, situated at 284.6 eV. The peak deconvolution was performed using Gaussian (70%) and Lorentzian (30%) (GL) functions within the CASA software, with Shirley background removal applied during the fitting process. The Cd 3d_5/2_ peak in Figure 5a appears at 404.5 eV for CZ900_Pris, CZ900_313O, and CZ900_313Ag thin films, indicative of Cd–O bonding [18–19]. Notably, the Cd 3d_5/2_ peak position for the CZ900_313Ag thin film has shifted toward lower binding energy. This shift can be attributed to the formation of V_O_ defects, resulting in reduced oxidation of the Cd atom, as reflected in the observed binding energy change. Figure 5b presents Zn 2p_3/2_ peaks for the three thin films. The Zn 2p_3/2_ peak is located at 1021.2 eV, corresponding to Zn–O bonds. Additionally, a low-intensity peak at 1021.7 eV in the CZ900_Pris thin film is associated with the willemite Zn_2_SiO_4_ phase. This peak is absent in the CZ900_313O and CZ900_313Ag thin films, likely due to lattice damage induced by Ag and O ion irradiation at the film surface. Furthermore, the Zn 2p_3/2_ peak in the CZ900_313Ag thin film exhibits a shift toward lower binding energy, consistent with the formation of V_O_ defects. The presence of the Zn_2_SiO_4_ phase is further supported by the O 1s and Si 2p_3/2_ peaks, as shown in Figure 5c,d, respectively. The O 1s peak for the CZ900_Pris thin film is deconvoluted into three components: O(i), O(ii), and O(iii), corresponding to the CdO, ZnO, and Zn_2_SiO_4_ phases, located at 530.0, 530.8, and 531.7 eV, respectively. In the CZ900_313O thin film, the peak area for the O(iii) component has diminished, indicating that the electronic energy loss from O ion irradiation affects the Zn_2_SiO_4_ phase. However, it is important to note that the peak cannot be solely attributed to the Zn_2_SiO_4_ phase, as the presence of defects such as V_O_ is also possible. The small shift in binding energy for V_O_ defects and Zn_2_SiO_4_ phases makes it difficult to distinguish them. For the CZ900_Pris thin film, the O(iii) component can be confidently assigned to the Zn_2_SiO_4_ phase, given that this film was annealed in a flowing oxygen atmosphere, which minimizes the potential for V_O_ defects at the surface. In contrast, the area corresponding to the V_O_ peak has increased in the CZ900_313Ag thin film. The generation of V_O_ in semiconducting oxides due to energetic ion irradiation is well-documented, as the formation energy for V_O_ is lower than that for defects such as cation interstitials [7]. The peak at 532.2 eV is thus solely attributed to V_O_, with no contribution from the Zn_2_SiO_4_, as indicated by the single peak fitting of the Si 2p_3/2_ peak in Figure 5d. The presence of the Zn_2_SiO_4_ phase is evident in the Si 2p_3/2_ peak for CZ900_Pris and CZ900_313O thin films. Therefore, the data from the O 1s and Si 2p_3/2_ peaks indicate that the Zn_2_SiO_4_ phase is not significantly affected by O ion irradiation. In contrast, Ag ion irradiation results in complete amorphization of the Zn_2_SiO_4_ phase at the film surface. The XPS spectra for the CZ900_Pris sample are reproduced from previous research [1].

### Track diameter calculation from inelastic thermal spike model

The phenomena of “Coulomb explosion” and “Thermal spike” represent two established models used to explain the high electronic-energy-loss-induced latent track and the subsequent amorphization effects resulting from SHI irradiation. In the first model, the formation of an ion track is attributed to the electrostatic repulsion among charged ions, which exceeds the chemical bond energy of the host lattice, ultimately leading to amorphization. The second model incorporates the concept of radial energy distribution to account for track formation. Within the framework of the inelastic thermal spike model, energy transfer to the electronic system occurs through electron–electron interactions, followed by transference to the lattice atomic system via electron–phonon correlation [20–21]. Along the ion trajectory, a cylindrical region is generated, characterized by temperature exceeding the melting point of the material, which facilitates the amorphization process. The confined of molten material within this narrow cylindrical volume promotes rapid cooling, thereby enhancing the quenching process and resulting in solidification and ion track formation. We have calculated the track diameter for CdO subjected to 120 MeV silver ion irradiation. The subsequent two coupled differential equations describe the energy distribution within the electronic and lattice subsystems, framed within cylindrical geometry, and represent the transient thermal process involved:


[2] $$C_{\text{e}}\frac{\partial T_{\text{e}}}{\partial t}=\nabla \left(K_{\text{e}}\nabla T_{\text{e}}\right)-g\left(T_{\text{e}}-T_{\text{a}}\right)+B\left(r,t\right),$$

[3] $$\rho C\left(T_{\text{a}}\right)\frac{\partial T_{\text{a}}}{\partial t}=\nabla \left(K_{\text{a}}\left(T_{\text{a}}\right)\nabla T\right)+g\left(T_{\text{e}}-T_{\text{a}}\right).$$

Equation 2 corresponds to the energy transfer to the electronic sub-system, and Equation 3 describes the same for the lattice subsystem. Here *C*_e_ and *C*(*T*_a_) are the specific heat; *T*_e_ and *T*_a_ are the temperatures; *K*_e_, and *K*_a_ are the specific heat values of the electronic and atomic subsystem, respectively. *g* is the electron–phonon coupling constant, and ρ is the specific mass of the lattice. In Equation 2, *B*(*r*,*t*) indicates the energy density provided to the electron subsystem by the SHI [22–23].

**Figure 6:**
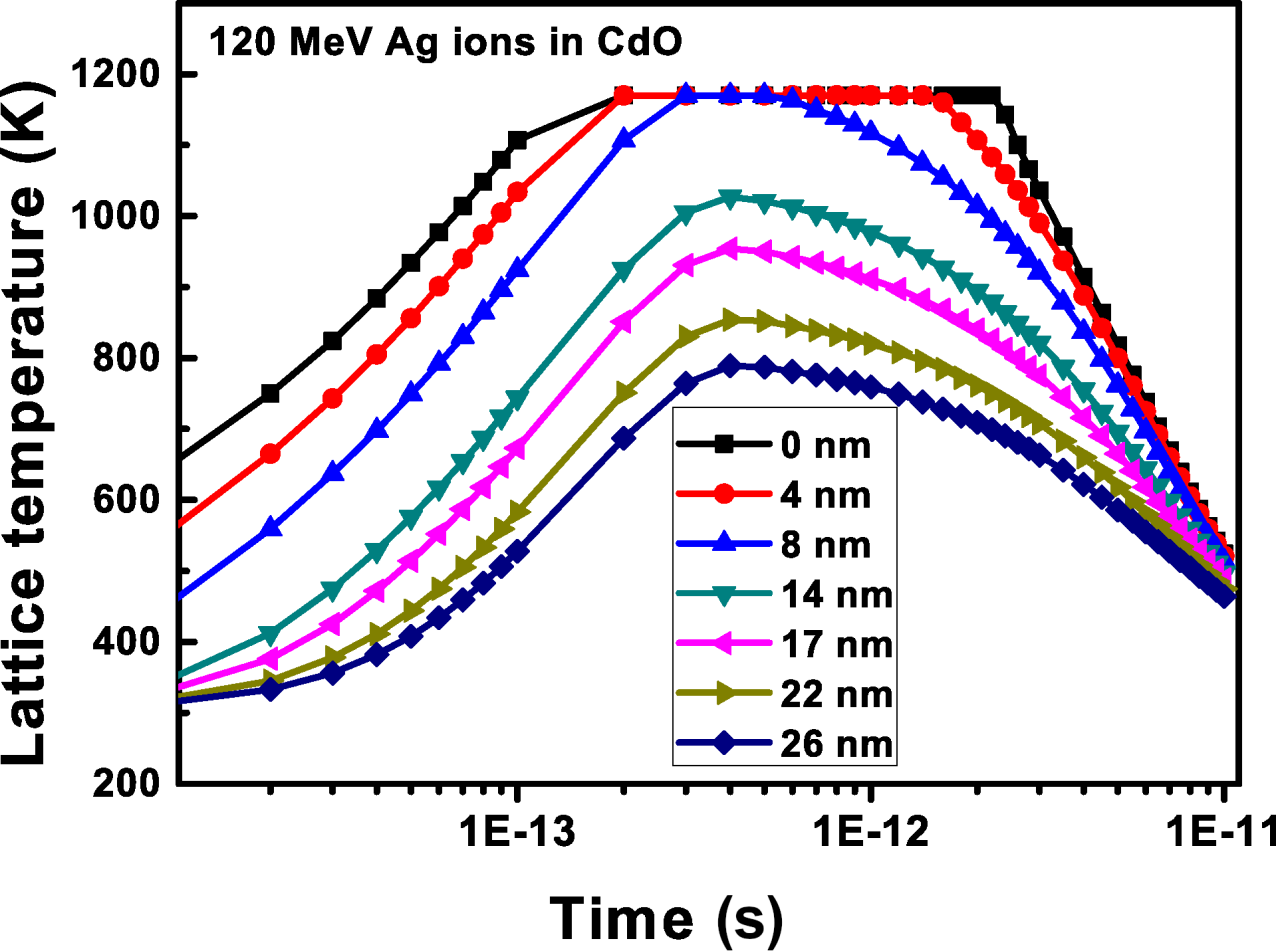
Plot showing lattice temperature as a function of time using thermal spike code to track diameter calculation.

The numerical solutions of the coupled differential equation, obtained through simulation codes, yield a graph depicting the temperature of the ion core as a function of time relative to the difference from the ion core, as shown in Figure 6. This analysis focuses on the calculated track diameter for cadmium oxide (CdO) subjected to 120 MeV silver ion irradiation. Figure 6 clearly indicates that the measured track diameter is 8 nm, corresponding to the melting temperature derived from multiple impact processes. Given that CdO has significantly high electrical conductivity (>10^14^ S/cm) and high mobility (>100 cm^2^/V/s), the simulation was conducted under the assumption that CdO behaves as a metallic system [2–4]. Furthermore, due to the minimal variation in lattice temperature (≈300 K) and Debye temperature (≈255 K) for CdO, the electron–phonon coupling constant can be expressed using the following equations:


[4] $$g=\frac{\pi^{4}{\left(k_{\text{B}}n_{\text{e}}v\right)}^{2}}{18k_{\text{e}}\left(T_{\text{e}}\right)},$$

where *v* is the velocity of sound in CdO, *n*_e_ is the electron number density, *K*_e_ and *T*_e_ are the specific heat and temperature of the electronic system, and


[5]

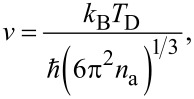



where *T*_D_ is the Debye temperature and *n*_a_ is the atomic number density. All the calculated values used in the simulation code are mentioned in Table 2.

**Table 2:** The calculated values used in the simulation code.

Bandgap (eV)	2.2
Solid density (g/cc)	8.15
Liquid density (g/cc)	6.95
Velocity of sound in CdO (m/sec)	2541.57
Electron–phonon coupling constant (W/cc-K)	1.38 × 10^13^
Se (keV/nm), SRIM 2013	25.1
Melting temperature (K)	1170
Lattice heat of fusion (Joule/g)	1896.27

### Discussion for retrieval of the B1 phase with O ion irradiation

The thermal annealing at temperatures exceeding 800 °C induces atomic interdiffusion at the interface between the Cd*_x_*Zn_1−_*_x_*O (*x* = 0.4) alloy film and the Si wafer substrate [24]. This process initiates significant diffusion of Si atoms, starting at the film–substrate interface and extending into the thin film layer, leading to the formation of Si–O bonds. At an annealing temperature of 900 °C, Si diffusion intensifies, resulting in an increased thickness of the amorphous silicon oxide layer at the film–substrate interface [24]. The interdiffusion of Si, O, Cd, and Zn atoms near the SiO*_x_* layer (i.e., at the substrate–film interface) facilitates the formation of willemite Zn_2_SiO_4_ nanoparticles [24]. Previous investigations have elucidated that the out-diffusion of Zn_2_SiO_4_ nanoparticles from the film–substrate interface to the surface occurs with a larger unit cell volume (1569.22 Å^3^) compared to CdO (105.29 Å^3^) and ZnO (47.40 Å^3^) generating localized pressure [1]. This localized pressure triggers the phase transition from B1 to B2 in CdO nanoparticles. Notably, 80 MeV oxygen ion irradiation results in the reappearance of the B1 phase, while 120 MeV silver ion irradiation completely amorphizes the B2 phase due to a higher *S*_e_ (25.1 keV/nm). In contrast, the *S*_e_ value for 120 MeV silver ions in ZnO is 1.62 keV/nm, indicating minimal impact on the wurtzite ZnO phase under silver ion irradiation. For Zn_2_SiO_4_, the *S*_e_ values for 80 MeV oxygen and 120 MeV silver ions are 8.2 keV/nm and 11.2 keV/nm, respectively, while the *S*_e_ value for 80 MeV oxygen ions in CdO is 1.9 keV/nm. All the energy loss values were calculated using the SRIM 2013 code. Consequently, the influence of oxygen ion irradiation is more pronounced in Zn_2_SiO_4_ than in CdO. This is attributable to the higher *S*_e_ value, which inflicts greater damage on the Zn_2_SiO_4_ lattice at the surface. The observed decrease in the intensity of the B2 phase peak and the reappearance of the B1 phase peak in the XRD patterns for the CZ900_113O and CZ900_313O films, as shown in Figure 1b, aligns with similar findings in Zn_2_SiO_4_. This phenomenon is attributed to a decrease in the local pressure exerted by Zn_2_SiO_4_ nanoparticles on CdO nanoparticles following oxygen ion irradiation at increasing ion fluences (3 × 10^13^ ions/cm^2^). The Zn *L*_3,2_ edge spectrum for CZ900_313O thin film in Figure 4b and the Si 2p_3/2_ peak in the XPS spectra in Figure 5d further corroborate this observation, indicating the presence of the Zn_2_SiO_4_ phase in oxygen-ion-irradiated thin films, while this phase is absent in those subjected to silver ion irradiation. The presence of the Zn_2_SiO_4_ nanoparticles in the oxygen-ion-irradiated films reaffirms the localized pressure exerted by these nanoparticles on CdO nanoparticles, causing the phase transition from B1 to B2. In the cases of the CZ900_113Ag and CZ900_313Ag thin films, both the willemite Zn_2_SiO_4_ nanoparticles and the B2 phase of CdO nanoparticles are entirely amorphized, leaving no possibility for the recovery of the B1 phase in these films. Thus, the primary reason for the reappearance of the B1 phase is the reduction of localized pressure exerted by Zn_2_SiO_4_ nanoparticles due to oxygen ion irradiation. This phase transition from B1 to B2 is reported to be irreversible under hydrostatic pressure [14] or localized pressure [1]. However, ion irradiation has emerged as an effective method to render this phase transition reversible, allowing for the subsequent recovery of the B1 phase at atmospheric pressure. From a thermodynamic perspective, the enthalpy difference (Δ*H*) in the phase transition process can be subdivided into two contributions: the pressure (Δ*PV*) and the change in internal energy (Δ*U*). The Δ*U* value is positive and independent of the pressure, serving as a barrier that stabilizes the B1 phase during the transition to the B2 phase. Transition to the B2 occurs when Δ*PV* surpasses this barrier. The increasing chemical pressure resulting from the diffusion of Zn_2_SiO_4_ nanoparticles renders Δ*PV* more negative than positive Δ*U*, thereby triggering the phase transition [25]. Consequently, for the pristine CZ900 thin film, Δ*PV* is more negative than positive Δ*U*. Following oxygen ion irradiation, the reduction of localized pressure on CdO nanoparticles results in Δ*PV* becoming less negative than positive Δ*U,* which further promotes the resurgence of the B1 phase.

## Conclusion

The presence of the willemite Zn_2_SiO_4_ phase was identified as a primary factor in generating localized pressure on CdO nanoparticles, facilitating the B1 to the B2 phase transition. This study investigated the irradiation stability of the B2 phase through SHI with 120 MeV silver and 80 MeV oxygen ions, revealing distinct behaviors in response to each ion type. Silver ion irradiation was shown to induce amorphization in the B2 phase, while oxygen ion irradiation led to the notable recovery of the B1 phase. This suggests that the electronic energy loss associated with oxygen ion irradiation partially damages the Zn_2_SiO_4_ lattice, resulting in the deterioration of local pressure on CdO nanoparticles and promoting the recovery of the B1 phase. The absence of the spike-like pre-edge feature in the Zn *L*_3,2_ edge with silver ion irradiation indicates the complete amorphization of the Zn_2_SiO_4_ phase. However, the intensity of this feature remains relatively unchanged with oxygen ion irradiation. The fitting analysis of the Si 2p XPS peak, showing the absence of the component related to Zn_2_SiO_4_ after Ag ion irradiation, further supports these findings. The correlation between core-level spectroscopic techniques and X-ray diffraction patterns reinforces the conclusion that electronic energy loss from oxygen ions leads to partial degradation of the Zn_2_SiO_4_ crystalline structure, diminishing local pressure on the CdO nanoparticles with the B2 phase and facilitating the retrieval of the B1 phase. Furthermore, the calculated track diameter for 120 MeV silver ions within the CdO rock salt phase was determined to be 8 nm. Collectively, these findings enrich the understanding of the B1 to B2 phase transition phenomenon and substantiate the hypothesis regarding local pressure-induced phase transitions mediated by the willemite Zn_2_SiO_4_ phase.

## Data Availability

All data that supports the findings of this study is available in the published article and/or the supporting information of this article.

## References

[1] Das A, Latouche C, Jobic S, Gautron E, Merabet A, Zajac M, Shibui A, Krüger P, Huang W-H, Chen C-L (2024). Acta Mater.

[2] Yu K M, Mayer M A, Speaks D T, He H, Zhao R, Hsu L, Mao S S, Haller E E, Walukiewicz W (2012). J Appl Phys.

[3] Yang Y, Jin S, Medvedeva J E, Ireland J R, Metz A W, Ni J, Hersam M C, Freeman A J, Marks T J (2005). J Am Chem Soc.

[4] Yan M, Lane M, Kannewurf C R, Chang R P H (2001). Appl Phys Lett.

[5] Schleife A, Rödl C, Furthmüller J, Bechstedt F (2011). New J Phys.

[6] Bharath S P, Bangera K V, Shivakumar G K (2017). J Alloys Compd.

[7] Das A, Singh F (2017). Vacuum.

[8] Lian J, Wang L M, Wang S X, Chen J, Boatner L A, Ewing R C (2001). Phys Rev Lett.

[9] Wang L, Gong W, Wang S, Ewing R C (1999). J Am Ceram Soc.

[10] Barbu A, Dunlop A, Lesueur D, Averback R S (1991). Europhys Lett.

[11] Liu L, Huang Y, Li Y, Fang L, Dammak H, Fan H, Thi M P (2012). Mater Lett.

[12] Szenes G (1995). Phys Rev B.

[13] Audouard A, Balanzat E, Bouffard S, Jousset J C, Chamberod A, Dunlop A, Lesueur D, Fuchs G, Spohr R, Vetter J (1990). Phys Rev Lett.

[14] Liu H, Mao H-k, Somayazulu M, Ding Y, Meng Y, Häusermann D (2004). Phys Rev B.

[15] Zając M, Giela T, Freindl K, Kollbek K, Korecki J, Madej E, Pitala K, Kozioł-Rachwał A, Sikora M, Spiridis N (2021). Nucl Instrum Methods Phys Res, Sect B.

[16] Szlachetko J, Szade J, Beyer E, Błachucki W, Ciochoń P, Dumas P, Freindl K, Gazdowicz G, Glatt S, Guła K (2023). Eur Phys J Plus.

[17] Gayen R N, Sarkar K, Hussain S, Bhar R, Pal A K (2011). Indian J Pure Appl Phys.

[18] Das A, Saini C P, Singh D, Ahuja R, Kaur A, Aliukov S, Shukla D, Singh F (2019). Nanoscale.

[19] Das A, Singh D, Saini C P, Ahuja R, Kaur A, Aliukov S (2020). Nanoscale.

[20] Valdez J A, Chi Z, Sickafus K E (2008). J Nucl Mater.

[21] Benyagoub A (2010). Nucl Instrum Methods Phys Res, Sect B.

[22] Wang Z G, Dufour C, Paumier E, Toulemonde M (1995). J Phys: Condens Matter.

[23] Toulemonde M, Dufour C, Wang Z, Paumier E (1996). Nucl Instrum Methods Phys Res, Sect B.

[24] Yuk J M, Lee J Y, Jung J H, Lee D U, Kim T W, Son D I, Choi W K (2008). J Appl Phys.

[25] Chen Y, Zhang S, Gao W, Ke F, Yan J, Saha B, Ko C, Suh J, Chen B, Ager J W (2016). Appl Phys Lett.

